# Early Post-Discharge Albuminuria Trajectory and Clinical Outcomes After Acute Heart Failure

**DOI:** 10.3390/jcm15155825

**Published:** 2026-07-25

**Authors:** Mario Galván-Ruiz, Patricia Nogueira-Salgueiro, Jonathan Déniz-Rosario, María del Val Groba-Marco, Miguel Fernández-de-Sanmamed-Girón, Jesús María González-Martín, Daesub Chung-Kwon, Belén Rojas-Escrivá, Marco Antonio Suarez-Benítez, Elvira Martín-Bou, Sara Aladro-Escribano, Juan Carlos Quevedo-Reina, Casimira Domínguez-Cabrera, Luis Burgos-Ramírez, Antonio García-Quintana, Alicia Conde-Martel

**Affiliations:** 1 Cardiology Department, Hospital Universitario de Gran Canaria Doctor Negrín, 35016 Las Palmas de Gran Canaria, Spain; 2 Health Sciences Faculty, Universidad de Las Palmas de Gran Canaria (ULPGC), 35016 Las Palmas de Gran Canaria, Spain; 3 Clinical Analysis Department, Hospital Universitario de Gran Canaria Doctor Negrín, 35010 Las Palmas de Gran Canaria, Spain; 4 Research Unit, Hospital Universitario de Gran Canaria Doctor Negrín, 35010 Las Palmas de Gran Canaria, Spain; 5 Nephrology Department, Complejo Hospitalario Universitario Insular, 35010 Las Palmas de Gran Canaria, Spain; 6 Nephrology Department, Hospital Universitario de Gran Canaria Doctor Negrín, 35010 Las Palmas de Gran Canaria, Spain; 7 Internal Medicine Department, Hospital Universitario de Gran Canaria Doctor Negrín, 35010 Las Palmas de Gran Canaria, Spain

**Keywords:** acute heart failure, urine albumin-to-creatinine ratio, congestion, vulnerable phase and biomarkers

## Abstract

**Background:** Albuminuria is a marker of cardiovascular and renal risk and may reflect dynamic haemodynamic changes in acute heart failure (AHF). However, the prognostic value of early urine albumin-to-creatinine ratio (UACR) monitoring during the vulnerable post-discharge phase remains unclear. **Methods:** We conducted a prospective single-centre study, including 88 patients with hospitalization for AHF. UACR was measured at discharge and at a two-week follow-up visit. Patients were stratified according to UACR trajectory: (1) persistent normoalbuminuria; (2) microalbuminuria with significant reduction (>50%) or normalization; and (3) persistent microalbuminuria without significant reduction or macroalbuminuria. Clinical outcomes included HF readmissions, all-cause mortality and a composite endpoint of HF readmission or all-cause mortality. **Results:** Albuminuria was prevalent in 64.8% of patients. Group 3 showed a higher burden of comorbidities, worse renal function and higher congestion markers. During a median follow-up of 553 days, group 3 had a higher incidence of HF events and the composite endpoint. In multivariable analysis, group 3 was independently associated with HF hospitalization (HR 3.09, 95% CI 1.19–8.04; *p* = 0.021) and the composite endpoint (HR 3.31, 95% CI 1.18–9.35; *p* = 0.024), whereas no independent association was observed for group 2. Renal function remained stable in groups 1 and 2, while group 3 showed worse renal outcomes. **Conclusions:** Albuminuria is frequently observed during AHF and is closely related to haemodynamic congestion. Persistent albuminuria identifies a high-risk phenotype, whereas early reduction was associated with more favourable unadjusted outcomes. Early post-discharge UACR trajectory may improve risk stratification during the vulnerable phase.

## 1. Introduction

Chronic kidney disease (CKD) frequently coexists with heart failure (HF) and contributes substantially to cardiovascular morbidity and mortality [1,2]. Albuminuria, assessed by the urine albumin-to-creatinine ratio (UACR), is an early marker of kidney damage and an established predictor of cardiovascular risk, even in patients with preserved estimated glomerular filtration rate (eGFR) [3,4].

In patients with HF, albuminuria is thought to result from multiple interacting mechanisms including endothelial dysfunction, systemic inflammation, neurohormonal activation, renal haemodynamic alterations and the burden of common comorbidities such as diabetes, hypertension and obesity [5,6]. In addition to structural renal injury, haemodynamic mechanisms related to systemic congestion may also contribute to albumin leakage through the glomerular filtration barrier [7,8]. Elevated central venous pressure increases renal venous pressure, impairs renal perfusion and promotes albumin excretion. This highlights the close relationship between congestion and renal dysfunction in HF [9].

Rather than representing a fixed indicator of CKD, albuminuria may also behave as a dynamic parameter that changes according to the patient’s haemodynamic status. Studies in acute heart failure (AHF) have shown that UACR decreases following effective decongestion, suggesting its potential role as a surrogate of reversible cardiorenal dysfunction during the acute phase [10,11].

However, the clinical implications of early changes in albuminuria after hospital discharge remain poorly characterized. The early post-discharge period, commonly defined as the vulnerable phase, is associated with a particularly high risk of HF rehospitalization and mortality [12,13]. Biomarkers capable of detecting early changes during this transition period may help refine risk stratification after hospital discharge.

Accordingly, this study aimed to characterize early changes in UACR between hospital discharge and the scheduled two-week post-discharge visit and to investigate whether different post-discharge UACR trajectories were associated with subsequent clinical and renal outcomes during follow-up, including HF readmission, all-cause hospitalization or emergency department visits, renal replacement therapy and all-cause mortality. In addition, we explored clinical predictors associated with the absence of a significant reduction in UACR trajectory and evaluated the potential prognostic implications of early changes in UACR during the vulnerable post-discharge phase.

## 2. Material and Methods

### 2.1. Study Design and Population

We performed an observational, single-centre, prospective study including eighty-eight consecutive patients admitted for acute heart failure (AHF) to the Cardiology Department of a tertiary care hospital.

Eligible patients were ≥18 years old with a clinical diagnosis of AHF according to the 2021 European Society of Cardiology guidelines, regardless of left ventricular ejection fraction (LVEF) [13]. To evaluate the trajectory of UACR between hospital discharge and the two-week post-discharge visit, only patients with available measurements at both time points were included in the final analysis.

Exclusion criteria included: (1) concomitant acute coronary syndrome; (2) requirement for renal replacement therapy prior to or during hospitalization; (3) incomplete blood or urine laboratory measurements; (4) in-hospital death; (5) absence of laboratory assessment at the two-week post-discharge visit; and (6) conditions known to significantly affect UACR levels, such as active infection, severe hyperglycaemia at admission (>500 mg/dL), or poorly controlled hypertension (systolic/diastolic blood pressure > 180/120 mmHg).

Blood and urine samples were collected at admission, at hospital discharge and during the scheduled two-week outpatient visit for biomarker assessment. Patients received treatment according to contemporary guideline-directed management and routine clinical practice throughout hospitalization. After discharge, follow-up was performed according to the institutional HF programme.

Baseline demographic information, cardiovascular risk factors, medical history, laboratory findings and echocardiographic variables were prospectively recorded. Clinical follow-up included assessment of HF readmission, emergency department visits, all-cause hospitalization and all-cause mortality. Renal outcomes comprised the need for renal replacement therapy together with longitudinal changes in eGFR and UACR.

**Figure 1:**
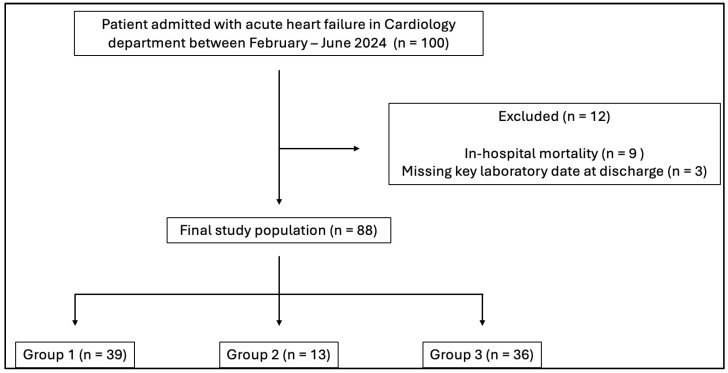
Study flowchart and patient classification according to early post-discharge UACR trajectory.

This study was conducted in accordance with the principles of Good Clinical Practice and the Declaration of Helsinki. The study protocol was approved by the local ethics committee (2023-150-1), and all patients provided informed consent for participation. A flow diagram summarizing patient selection is shown in Figure 1.

### 2.2. Grading and Scoring Clinical Congestion

Clinical congestion was assessed using the Clinical Congestion Score (CCS) [14]. Based on the predominant clinical presentation at admission, patients were categorized according to congestion phenotype in right-sided congestion phenotype and left-sided congestion phenotype. Individuals presenting with both right and left-sided features were categorized as having a mixed congestion phenotype.

### 2.3. Laboratory Analysis

Urine and blood samples were collected at hospital discharge and at the scheduled two-week post-discharge visit. Biomarkers assessed included UACR, N-terminal pro-B-type natriuretic peptide (NT-proBNP) and serum cancer antigen 125 (CA125).

Based on the evolution of UACR between both assessments, patients were classified into three predefined trajectory groups: (1) normoalbuminuria (<30 mg/g) at both time points; (2) microalbuminuria (30–300 mg/g) at discharge with either a >50% reduction in UACR or normoalbuminuria at two weeks; and (3) microalbuminuria at discharge with <50% reduction at two weeks or the presence of macroalbuminuria (>300 mg/g) at either time point.

The >50% reduction threshold was selected as a pragmatic criterion to identify a clinically meaningful decrease in albuminuria beyond expected biological variability and measurement error. This threshold is also supported by previous analyses of the TOPCAT trial, in which a 50% reduction in UACR was associated with improved clinical outcomes [5,15].

Additional laboratory variables included renal function parameters, liver function tests, urinary sodium and chloride concentrations and hemoglobin levels.

### 2.4. Study Endpoint

The primary endpoint was the composite of HF readmission or all-cause mortality during follow-up. Secondary endpoints included HF readmission, all-cause mortality, emergency department visits, all-cause hospitalizations and renal outcomes, including renal replacement therapy and longitudinal changes in eGFR and UACR.

The primary study objective was to evaluate whether early post-discharge UACR trajectory was associated with these clinical outcomes.

As an exploratory (hypothesis-generating) analysis, we evaluated whether early implementation of guideline-directed medical therapy (GDMT) at discharge in patients with LVEF < 50% was associated with the composite endpoint.

### 2.5. Statistical Analysis

Continuous variables were expressed as mean ± standard deviation (SD) when normally distributed or as median and interquartile range (IQR) when non-normally distributed, and categorical variables as frequencies and percentages.

Comparisons between categorical variables were performed using the Chi-square test or Fisher’s exact test, as appropriate. For continuous variables, Student’s test-or the Mann–Whitney U test were used for comparison between two groups and one-way ANOVA or the Kruskall-Wallis test for comparisons across three groups, as appropriate.

Paired comparisons between measurements obtained at hospital discharge and at the two-week follow-up visit were performed using paired Student’s *t*-test or the Wilcoxon signed-rank test.

Time-to-event analyses were performed using Cox proportional hazards regression models to evaluate the association between UACR trajectory groups and clinical outcomes. For the analysis of HF hospitalization, patients who died before experiencing the event were censored at the time of death. Variables considered clinically relevant and/or showing a *p*-value < 0.10 in univariable analysis were considered for inclusion in the multivariable Cox models. To minimize the risk of overfitting, the number of covariates included in each model was restricted according to the number of outcome events. The events per variable (EPV) were calculated for each multivariable model, and model optimism was assessed by bootstrap internal validation (1000 resamples); a parsimonious model including only trajectory group and previous HF hospitalization was additionally fitted as a sensitivity analysis.

Event-free survival was estimated using Kaplan–Meier survival curves and compared with the log-rank test. Hazard ratios (HR) with 95% confidence intervals (CI) were reported. The proportional hazards assumption was tested.

Predefined sensitivity analyses were performed to evaluate the robustness of the main findings. These included repeating the primary Cox regression analyses after excluding patients with baseline macroalbuminuria and reclassifying UACR trajectory using alternative reduction thresholds.

All tests were two-tailed, and a *p*-value < 0.05 was considered statistically significant. Statistical analyses were conducted using Jamovi (version 2.3.21.0) and RStudio (version 4.4.2).

### 2.6. Patient and Public Involvement Statement

Patients and members of the public were not involved in the design, conduct, reporting, or dissemination plans of this study.

## 3. Results

A total of 88 patients were included, with a mean age of 70.2 ± 12.5 years (range 34–94 years), of whom 43.2% were women. The median follow-up was 553 days (IQR 289–618). The most common comorbidities were hypertension (72.7%), dyslipidaemia (60.2%), atrial fibrillation (54.5%), diabetes mellitus (53.4%) and CKD (37.5%). Previous HF hospitalizations were reported in 34.1% of patients.

Regarding HF GDMT at admission, 45.5% of patients were receiving beta-blockers, 53.8% angiotensin-converting enzyme inhibitors or angiotensin II receptor blockers (ACEI/ARB), 12.5% angiotensin receptor–neprilysin inhibitors (ARNI), 21.6% mineralocorticoid receptor antagonists (MRA), 33% sodium–glucose cotransporter-2 inhibitors (SGLT2i) and 40% loop diuretics. The median CCS at admission was 5 (IQR 4–5).

The median LVEF was 40% (IQR 32–55), the left ventricular end-diastolic diameter (LVEDD) was 55 mm (IQR 48–60) and the TAPSE/systolic pulmonary artery pressure (PSAP) ratio was 0.44 (IQR 0.29–0.63).

At admission, the median eGFR was 56 mL/min/1.73 m^2^ (IQR 38–78) and the median UACR was 67.9 mg/g (IQR 17.6–209). According to baseline UACR levels, patients were classified as normoalbuminuria (*n* = 31, 35.2%), microalbuminuria (*n* = 38, 43.2%) and macroalbuminuria (*n* = 19, 21.6%). Other biomarkers included NT-proBNP with a median value of 3676 pg/mL (IQR 2357–7191) and CA125 of 73.4 U/mL (IQR 31–165).

### 3.1. Baseline Characteristics According to UACR Groups (Discharge—Two Weeks Post-Discharge)

Patients were classified into three groups according to UACR trajectory between discharge and two-week follow-up visit: group 1 (44.3%, *n* = 39), group 2 (14.8%, *n* = 13) and group 3 (40.9%, *n* = 36) (Table 1).

Patients in group 3 had a higher burden of comorbidities, including dyslipidaemia and CKD, with a trend toward a higher prevalence of hypertension, diabetes mellitus and atrial fibrillation. They also had more previous HF hospitalizations, higher UACR levels at admission and higher CCS values during the admission.

Patients in group 3 more frequently received MRA and SGLT2i before admission. At hospital discharge, they required higher loop diuretic doses and showed a trend toward greater thiazides use, whereas no differences were observed in GDMT.

At discharge, group 3 had worse renal function, higher alkaline phosphatase levels and higher NT-proBNP levels. These differences persisted at the two-weeks post-discharge visit, together with a trend toward higher CA125 levels and lower urinary sodium and chloride levels.

Echocardiographic findings showed similar LVEF across groups; however, group 3 showed higher pulmonary hypertension, a worse TAPSE/PSAP ratio and more severe valvular disease.

### 3.2. Clinical Outcomes According to UACR Trajectory

Patients in group 3, experienced significantly more HF events during follow-up, including hospitalizations or emergency department visits due to HF, as well as a higher incidence of the composite endpoint of all-cause mortality or HF readmission (Table 2).

Notably, all HF-related deaths (*n* = 6) occurred in patients belonging to group 3. A trend toward a higher rate of non-cardiovascular hospitalizations was also observed in this group.

### 3.3. Renal Outcomes According to UACR Trajectory

Nine patients died before completing one year of follow-up and were therefore excluded from the renal outcome analysis. At the end of follow-up, one patient from group 1, two from group 2, and six from group 3 had died. Notably, all patients who required renal replacement therapy during follow-up belonged to group 3.

**Figure 2:**
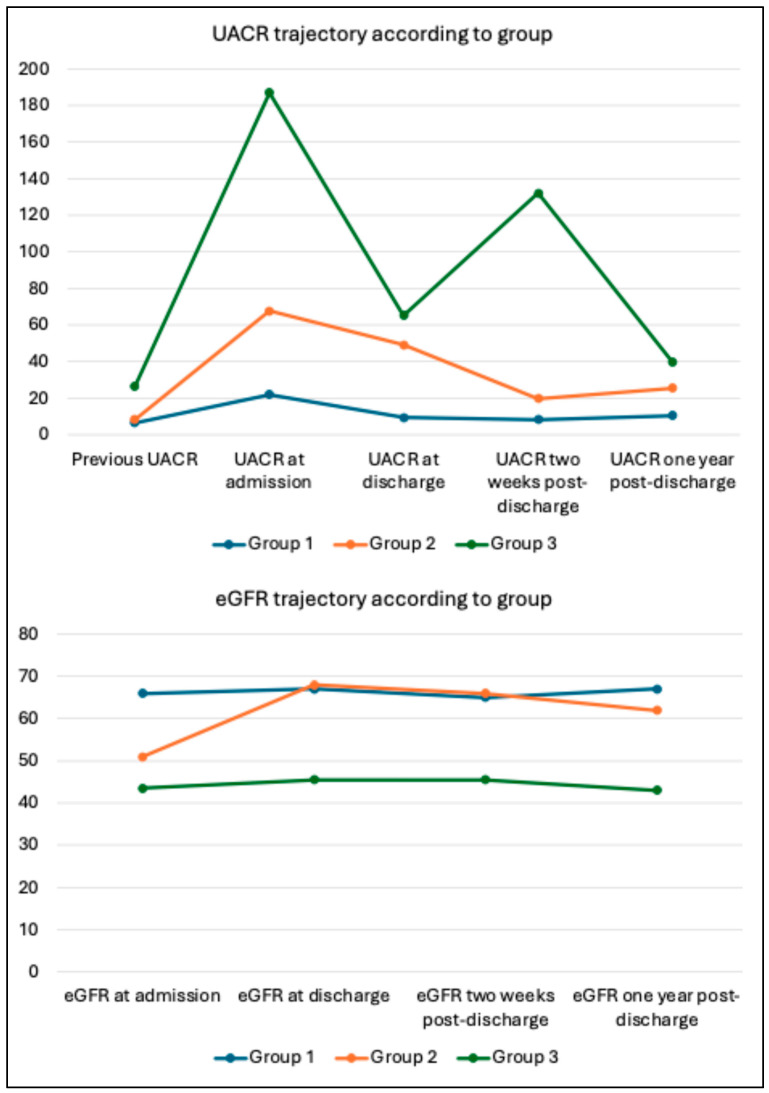
Longitudinal changes in UACR and eGFR according to albuminuria trajectory groups from admission to follow-up.

Figure 2 illustrates the longitudinal evolution of UACR and eGFR according to trajectory groups. The median UACR in groups 1 and 2 remained below 30 mg/g at one-year follow-up, whereas in group 3 the median UACR remained above 30 mg/g (*p* = 0.050). Although this difference did not reach statistical significance, it showed a trend toward persistently higher albuminuria despite optimization of medical therapy.

Regarding renal function, eGFR remained higher and relatively stable in groups 1 and 2 during follow-up. In contrast, a slight decrease in eGFR was observed in group 3 compared with baseline; however, this change did not reach statistical significance (*p* = 0.073).

### 3.4. Clinical Outcomes According to Composite Endpoint

Univariable analysis was performed to identify clinical factors associated with the composite endpoint (Supplementary Table S1, showing the baseline characteristics of patients according to the occurrence of the composite endpoint).

A higher incidence of the composite endpoint was observed in patients belonging to groups 2 and 3. In contrast, after multivariable adjustment, only group 3 remained independently associated with the composite endpoint. Patients with events were older and had a higher burden of comorbidities.

They also had more previous HF hospitalizations and more frequently required intensified diuretic therapy during the index admission.

At discharge, these patients received higher doses of loop diuretics and showed a lower use of ARNI and SGLT2i.

They also showed worse renal function and higher NT-proBNP levels at discharge, which persisted at follow-up, together with lower urinary sodium and chloride levels.

From an echocardiographic perspective, patients who experienced events during follow-up showed a trend toward higher LVEF, with a higher proportion of HF with preserved ejection fraction (HFpEF). They also presented higher PASP, a worse TAPSE/PASP ratio and a higher prevalence of significant tricuspid regurgitation.

### 3.5. Multivariable Cox Regression Analysis

Multivariable Cox regression models were used to identify predictors of HF hospitalization and the composite endpoint.

**Figure 3:**
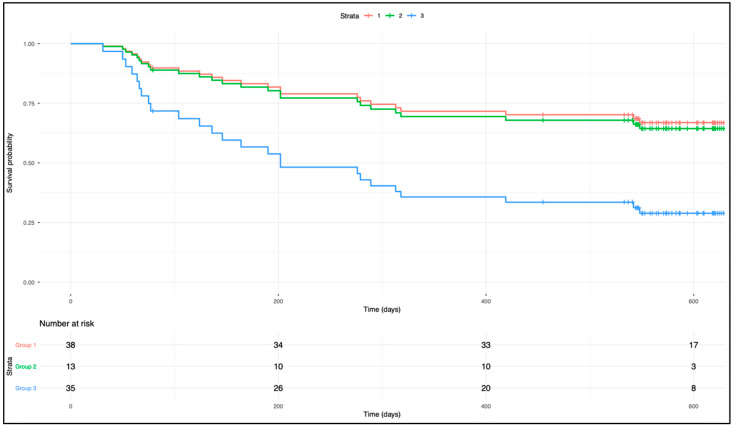
Kaplan–Meier curves for heart failure hospitalization according to early post-discharge UACR trajectory groups. Comparisons between groups were performed using the log-rank test. Time since hospital discharge is expressed in days. Numbers at risk are shown below the graph.

In the optimal multivariable model for HF hospitalizations (Supplementary Table S2, showing the univariable and multivariable Cox regression analyses for HF hospitalization), group 3 was independently associated with higher risk compared with group 1 (HR 3.09, 95% CI 1.19–8.04; *p* = 0.021), together with previous HF hospitalization and LVEF (Figure 3).

**Figure 4:**
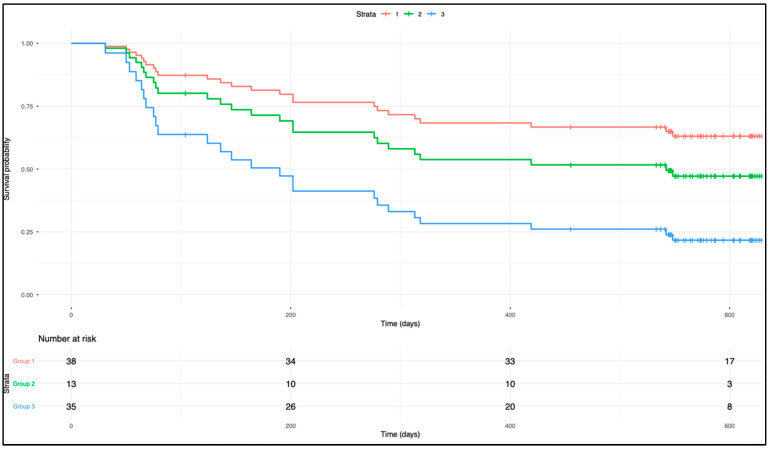
Kaplan–Meier curves for the composite endpoint (heart failure hospitalization or all-cause mortality) according to early post-discharge UACR trajectory groups. Comparisons between groups were performed using the log-rank test. Time since hospital discharge is expressed in days. Numbers at risk are shown below the graph.

Similarly, in the multivariable model for the composite endpoint (Supplementary Table S3, showing the univariable and multivariable Cox regression analyses for the composite endpoint), group 3 remained independently associated with a higher risk of events (HR 3.31, 95% CI 1.18–9.35; *p* = 0.024), along with older age, previous HF hospitalization and LVEF (Figure 4).

Given the limited number of events, the events per variable (EPV) were 5.8 for the composite endpoint model and 6.8 for the HF hospitalization model. Bootstrap internal validation (1000 resamples) indicated moderate optimism for both models (composite endpoint: apparent C-index 0.778, optimism-corrected 0.746, shrinkage factor 0.821; HF hospitalization: 0.762, 0.729, 0.783; Supplementary Table S4). In a parsimonious model including only trajectory group and previous HF hospitalization (EPV 9.7 and 9, respectively), group 3 remained independently associated with both the composite endpoint (HR 2.49, 95% CI 1.02–6.10) and HF hospitalization (HR 2.49, 95% CI 1.01–6.10), consistent in direction and magnitude with the primary models.

### 3.6. Sensitivity Analysis

To evaluate whether the observed prognostic association was driven by patients with baseline macroalbuminuria, we performed an additional sensitivity analysis excluding the five patients with macroalbuminuria (>300 mg/g) present at hospital discharge. Patients who developed macroalbuminuria between discharge and the two-week visit were retained in Group 3, as this progression represented the unfavourable UACR trajectory under investigation. After excluding patients with baseline macroalbuminuria, Group 3 remained associated with the composite endpoint, although it was of borderline statistical significance (HR 2.61, 95% CI 1.00–6.83; *p* = 0.050). Similarly, the association with HF hospitalization remained significant (HR 2.92, 95% CI 1.10–7.74; *p* = 0.032), supporting the primary findings (Supplementary Table S5, sensitivity analysis).

Additional sensitivity analyses using alternative UACR reduction thresholds (30%, 40%, 50%, 60%, and 70%) showed consistent results, with Group 3 remaining independently associated with both the composite endpoint and HF hospitalization across all thresholds, whereas no statistically significant differences were observed for Group 2 (Supplementary Table S6, detailing the results of the sensitivity analyses using alternative UACR reduction thresholds).

## 4. Discussion

Persistent albuminuria during the early post-discharge phase after AHF was associated with a high-risk cardiorenal phenotype characterized by a higher incidence of HF readmissions, adverse renal outcomes and worse long-term prognosis.

Albuminuria is a common finding in patients hospitalized for AHF. Previous studies have reported a prevalence of 82.6% in the TACRAHF cohort [10], around 69% in a Japanese cohort [11], compared with 64.8% in our study population. Beyond its high prevalence, albuminuria appears to be a dynamic biomarker that may improve following effective decongestion. UACR may increase during episodes of congestion (“wet value”) and decrease after decongestion (“dry value”), suggesting its potential role as a simple indicator of the haemodynamic status of patients with HF [16].

However, the clinical significance of UACR changes after discharge, particularly during the vulnerable post-discharge phase, remains poorly characterized.

Albuminuria is a well-established marker of cardiovascular and renal risk [17]. Previous studies have shown that higher UACR levels at admission are associated with worse outcomes and may provide incremental prognostic information beyond traditional biomarkers such as NT-proBNP, although data on post-discharge trajectory remain limited [18,19]. To our knowledge, prospective data evaluating the prognostic implications of early post-discharge UACR trajectory after AHF hospitalization remain scarce.

In our study, patients with persistent albuminuria or without a > 50% reduction in UACR (group 3) experienced a higher incidence of cardiovascular events, including HF readmissions and all-cause mortality during follow-up. These findings suggest that early UACR trajectory, rather than its absolute value alone, carries important prognostic significance. Patients who achieved normalization or a significant reduction in UACR showed a markedly better clinical course, whereas persistent albuminuria identified a high-risk phenotype. The consistency of the findings across multiple alternative thresholds supports the robustness of the proposed classification. Importantly, this association persisted in a sensitivity analysis excluding patients with baseline macroalbuminuria, remaining significant for HF hospitalization and of borderline significance for the composite endpoint, suggesting that the prognostic value of and unfavourable UACR trajectory was not exclusively driven by severe baseline albuminuria.

All patients requiring renal replacement therapy belonged to group 3, which also showed persistently impaired renal function, reinforcing the association between persistent UACR elevation and cardiorenal progression [20].

Patients in group 3 showed features of persistent congestion, including higher CCS, CA125 levels and higher NT-proBNP both at discharge and at the two-week visit. In addition, these patients required higher loop diuretic doses at discharge and greater use of thiazides, which may suggest incomplete decongestion despite intensified diuretic therapy. Therefore, persistent albuminuria may reflect residual congestion during the vulnerable post-discharge phase and provide a mechanistic explanation for the adverse cardiorenal outcomes observed in this subgroup. The pathophysiology of albuminuria in HF is multifactorial, involving glomerular injury, endothelial dysfunction, inflammation and haemodynamic congestion. Elevated venous pressure may impair renal perfusion and promote albumin leakage through the glomerular filtration barrier, providing a plausible mechanistic link between persistent congestion and sustained albuminuria [8]. Previous studies have suggested that albuminuria behaves as a dynamic congestion-related biomarker in acute heart failure, increasing during the “wet phase” and decreasing after decongestion, with trajectories paralleling NT-proBNP and CA125 changes [21,22].

In multivariable analysis, group 3 remained independently associated with adverse outcomes, whereas the association observed in group 2 did not remain statistically significant after adjustment. Although this subgroup had fewer HF readmissions, mortality was similar to that of group 3, and the small number of patients and events limited the statistical power to detect an independent association; this finding should therefore be regarded as inconclusive rather than as evidence of a comparable prognosis. Therefore, the apparent favourable prognosis associated with early UACR reduction should be interpreted with caution and considered hypothesis-generating until confirmed in larger prospective cohorts. Previous HF hospitalization was also an independent predictor.

A higher proportion of HFpEF was observed in group 3 (Supplementary Material Table S1), suggesting that albuminuria may be particularly relevant in this phenotype characterized by congestion and high burden of comorbidities [22,23].

Despite the small sample size, patients in group 3 exhibited lower urinary sodium and chloride levels at two weeks post-discharge. Previous studies and clinical trials (ENACT-HF [24] and PUSH-AHF [25]) have highlighted the role of natriuresis as an early marker of diuretic response. In contrast, evidence in the post-discharge setting remains limited, although lower urinary sodium has been associated with worse outcomes [26]. More recently, increasing attention has been directed toward the complementary role of chloride in heart failure, as chloride homeostasis appears to influence neurohormonal activation, diuretic response, and congestion status, supporting the clinical relevant of assessing urinary electrolytes beyond sodium alone [27].

In this context, our findings suggest that reduced urinary sodium may reflect diuretic resistance and persistent congestion during the vulnerable post-discharge phase [16]. This is further supported by higher NT-proBNP and CA125 levels observed in these patients, as well as the need for higher loop diuretic doses and greater use of thiazides [28]. Whether UACR provides incremental prognostic information beyond urinary electrolyte measurements or renal function cannot be determined from the present study. Rather, our findings suggest that these markers may reflect complementary pathophysiological processes related to persistent congestion and cardiorenal dysfunction, a hypothesis that warrants evaluation in larger prospective studies.

These findings suggest that early UACR trajectory after AHF hospitalization may provide a simple, inexpensive and widely available tool for risk stratification during the vulnerable post-discharge phase, potentially identifying patients who require more intensive follow-up and therapeutic optimization. Although the association remained significant after multivariable adjustment, residual confounding related to baseline differences in disease severity and congestion cannot be completely excluded. Notably, although patients in Group 3 more frequently received SGLT2 inhibitors before admission, their use was comparable across groups at discharge as was mineralocorticoid receptor antagonist use. This makes differential prescription of these therapies at discharge less likely to explain the observed UACR trajectories. Persistent albuminuria in Group 3 may therefore reflect, at least in part, a more advanced cardiorenal phenotype. Therefore, persistent albuminuria may represent an integrative marker of a more advanced cardiorenal phenotype, reflecting the combined effects of renal dysfunction, haemodynamic congestion and structural cardiac disease, endothelial dysfunction and chronic inflammatory activation, rather than a fully independent prognostic biomarker [29]. Whether albuminuria represents merely a marker of disease severity or plays a direct pathophysiological role in cardiorenal progression remains to be fully elucidated.

This study has several limitations. First, its single-centre design and relatively small sample size may limit the generalizability of the findings and may have limited the statistical power to detect some clinically relevant associations, particularly in group 2. In addition, the limited number of outcome events (29 for the composite endpoint and 27 for HF hospitalization, corresponding to 5.8 and 6.8 events per variable, respectively) may have increased the risk of overfitting in the multivariable Cox models. Bootstrap internal validation indicated moderate optimism, and the effect estimates should accordingly be interpreted with appropriate caution. Furthermore, the small number of patients in Group 2 limited the statistical power to establish whether an early reduction in UACR was independently associated with outcomes. Second, the observational nature of the study precludes establishing causal relationships and residual confounding related to baseline differences in disease severity congestion, renal dysfunction and treatment between groups cannot be completely excluded despite multivariable adjustment. In addition, patient management reflected routine clinical practice, which may have introduced variability in therapeutic strategies. Medication changes between hospital discharge and the first scheduled follow-up visit were not systematically recorded. Although major treatment modifications before this early reassessment were unlikely in routine clinical practice, their potential influence on UACR trajectory cannot be completely excluded. Baseline UACR reference values were not available for all patients, potentially limiting the interpretation of longitudinal changes. Finally, although serial NT-proBNP and CA 125 measurement and urinary electrolyte concentration were available, congestion assessment was mainly clinical and not supported by systematic objective techniques, which may have introduced interobserver variability in congestion status and should be considered when interpreting the observed associations. Future multicentre studies with larger populations, standardized congestion assessment, predefined therapeutic protocols, and advanced statistical approaches are warranted to better clarify the prognostic role of UACR trajectory after AHF and to determine whether albuminuria represents merely a marker of disease severity or a potentially modifiable pathophysiological HF pathway. Such studies will also be essential to validate the clinical utility of early UACR trajectory before its routine implementation in clinical practice.

## 5. Conclusions

Albuminuria is frequently observed during AHF hospitalization and is closely related to haemodynamic congestion. Patients with persistent albuminuria or without a significant reduction (>50%) represent a high-risk phenotype, with increased cardiovascular and renal events, HF readmissions and worse renal progression. These findings support the role of albuminuria as a dynamic biomarker of cardiorenal status, with potential utility for early risk stratification during the vulnerable post-discharge phase.

## Figures and Tables

**Table 1:** Baseline characteristics of the study population according to UACR trajectory group.

	Group 1 *N* = 39 (44.3%)	Group 2 *N* = 13 (14.8%)	Group 3 *N* = 36 (40.9%)	*p* Value
Women, *n* (%)	17 (43.6%)	6 (46.2%)	15 (41.7%)	0.959
Age (mean ± sd)	68.4 ± 12.3	73.1 ± 16	71.1 ± 11.5	0.311
BMI (mean ± sd)	28.2 ± 5.7	25.6 ± 3.96	28.6 ± 6.2	0.300
Follow-up (days, median (IQR)	586 (546–627)	550 (455–573)	542 (193–580)	0.024
Comorbidities				
Hypertension, *n* (%)	24 (61.5%)	10 (76.9%)	30 (83.3%)	0.099
Diabetes mellitus, *n* (%)	25 (64.1%)	5 (38.5%)	17 (47.2%)	0.173
Dyslipidaemia, *n* (%)	17 (43.6%)	9 (69.2%)	27 (75%)	0.016
COPD, *n* (%)	1 (2.6%)	2 (15.4%)	3 (8.3%)	0.254
Atrial fibrillation, *n* (%)	25 (64.1%)	8 (61.5%)	15 (41.7%)	0.129
CKD, *n* (%)	8 (20.5%)	4 (30.8%)	21 (58.3%)	0.003
Albuminuria				
Previous UACR, mg/g, median (IQR)	6.6 (3.9–13.2)	8.2 (5.8–14.5)	26.4 (13–146)	<0.001
UACR at admission, mg/g, median (IQR)	21.9 (8.1–81.4)	67.6 (29.3–128)	187 (45.7–356)	<0.001
<30 mg/g, *n* (%)	20 (51.3%)	4 (30.8%)	7 (19.4%)	0.015
30–300 mg/g, *n* (%)	17 (43.6%)	6 (46.2%)	15 (41.7%)	0.959
>300 mg/g, *n* (%)	2 (5.1%)	3 (23.1%)	14 (38.9%)	0.002
Clinical characteristics:				
Ischemic etiology, *n* (%)	10 (25.6)	2 (15.4)	9 (25)	0.738
Previous HF admission, *n* (%)	8 (20.5)	4 (30.8)	18 (50)	0.026
Right or mixed congestion phenotype, *n* (%)	13 (33.3)	6 (46.2)	18 (50)	0.326
Thiazides/acetazolamide/hypertonic saline use during admission, *n* (%)	7 (17.9)	3 (23.1)	6 (16.7)	0.875
Clinical congestion score, median (IQR)	4 (4–5)	5 (4–7)	5 (4–6)	0.021
Treatment before admission:				
Beta-blockers, *n* (%)	12 (30.8)	8 (61.5)	20 (55.6)	0.044
ACEI/ARB, *n* (%)	16 (59.3)	1 (25)	11 (52.4)	0.433
ARNI, *n* (%)	3 (7.7)	0	8 (22.2)	0.055
MRA, *n* (%)	4 (10.3)	2 (15.4)	13 (36.1)	0.021
SGLT2i, *n* (%)	8 (20.5)	3 (23.1)	18 (50)	0.018
Loop diuretics, *n* (%)	13 (33.3)	6 (46.2)	21 (58.3)	0.094
Dose Loop diuretics, (mean ± sd)	11 ± 18.7	18.5 ± 20.8	30 ± 39.3	0.046
Treatment at discharge:				
Beta-blockers, *n* (%)	36 (92.3)	12 (92.3)	30 (83.3)	0.427
ACEI/ARB, *n* (%)	8 (20.5)	4 (30.8)	7 (19.4)	0.680
ARNI, *n* (%)	29 (74.4)	8 (61.5)	21 (58.3)	0.322
MRA, *n* (%)	30 (76.9)	7 (53.8)	27 (75)	0.250
SGLT2i, *n* (%)	35 (89.7)	12 (92.3)	33 (91.7)	0.942
GLP1 receptor agonists, *n* (%)	2 (5.1)	1 (7.7)	2 (5.6)	0.941
Loop diuretics, *n* (%)	32 (82.1)	11 (84.6)	33 (91.7)	0.470
Dose Loop diuretics (mean ± sd)	33.7 ± 25.9	24.6 ± 14.5	47.4 ± 32.9	0.055
Thiazide use, *n* (%)	1 (2.6)	0	5 (13.9)	0.087
GDMT + LVEF < 50%, n (%)	20 (51.3%)	3 (23.1%)	15 (41.7%)	0.200
Laboratory parameters at discharge				
Urea (mg/dL) mg/dL, median (IQR)	48 (37–60)	53 (42–65)	83 (51–112)	<0.001
Creatinine mg/dL, median(IQR)	1.08 (0.89- 1.30)	1.13 (0.92–1.22)	1.34 (0.92- 2.03)	0.004
eGFR ml/min, median (IQR)	67 (50–83)	68 (60–77)	45.5 (28.3–64.3)	0.002
Sodium mEq/L, median (IQR)	138 (136–140)	137 (135–139)	137 (136–138)	0.257
Potassium mEq/L, median (IQR)	4.35 (4.14–4.68)	4.82 (4.66–4.95)	4.55 (4.28–4.96)	0.040
Chloride mEq/L, median (IQR)	101 (98–102)	101 (97–102)	98 (97–100)	0.139
AST U/L, median (IQR)	24 (17–33)	21 (17–31)	21 (16–25)	0.607
ALT U/L, median(IQR)	21 (14–30)	23 (14–69)	15 (12–23)	0.182
LDH, U/L, median (IQR)	229 (197–267)	273 (261–341)	224 (201–269)	0.070
Bilirubin, mg/dL, median (IQR)	0.51 (0.39–0.68)	0.44 (0.38- 0.62)	0.54 (0.34–0.73)	0.939
ALP, U/L, median (IQR)	68 (57–88)	85 (66–109)	89 (76–128)	0.008
GGT, U/L, median (IQR)	23 (19–41)	39 (27–49)	38 (19–76)	0.542
Troponin T, ng/L, median (IQR)	52 (26–70)	68 (42–110)	60 (28–125)	0.780
NT-proBNP, pg/mL, median (IQR)	1329 (675–2702)	2045 (1753–3761)	2704 (1290–8256)	0.016
CA 125, U/mL, median (IQR)	46.4 (23.5–142)	96.6 (44.6–234)	41.2 (31.6–114)	0.263
Urinary Sodium, mEq/L, median (IQR)	55.4 (25.7–63.9)	39.8 (24.6–50.9)	67.2 (42.8–84.2)	0.656
Urinary Chloride, mEq/L, median (IQR)	40.7 (19.7–49.4)	43.6 (24.1–66)	29.1 (17.3–45)	0.388
Hemoglobin, g/L, median (IQR)	11.9 (10.9–13.6)	12.8 (10.9–13.8)	12.6 (10.3–14.2)	0.979
Laboratory parameters at two weeks post discharge				
Urea, mg/dL, median (IQR)	43 (37–57)	52 (43–67)	74 (44–100)	<0.001
Creatinine, mg/dL, median (IQR)	1.10 (0.90–1.21)	1.08 (0.96- 1.22)	1.38 (1.15- 2.04)	<0.001
eGFR, ml/min/1.73 m^2^, median (IQR)	65 (53–80)	66 (55–74)	46 (29–57)	<0.001
Sodium, mEq/L, median (IQR)	140 (138–141)	140 (137–141)	139 (136–142)	0.587
Potassium, mEq/L, median (IQR)	4.43 (4.13–4.82)	4.56 (4.29–5.12)	4.83 (4.29–5.07)	0.106
Chloride, mEq/L, median (IQR)	102 (100–104)	100 (98–102)	100 (97–103)	0.091
AST, U/L, median (IQR)	20 (16–27)	19 (16–22)	23 (19–28)	0.263
ALT, U/L, median (IQR)	19 (14–30)	16 (12–24)	17 (12–21)	0.283
LDH, U/L, median (IQR)	195 (175–224)	267 (202–297)	232 (198–295)	0.005
Bilirubin, mg/dL, median (IQR)	0.47 (0.38–0.75)	0.42 (0.33–0.51)	0,60 (0.42–0.76)	0.117
ALP, U/L, median (IQR)	86 (66–105)	87 (76–100)	98 (85–138)	0.077
GGT U/L, median (IQR)	26 (16–53)	29 (18–41)	37 (22–94)	0.165
NT-proBNP, pg/mL, median (IQR)	1114 (600–2282)	2589 (1121–3669)	3285 (1475–7382)	0.001
CA 125 U/mL, median (IQR)	29.4 (14.5–60.5)	30.2 (21.2–85.2)	42.2 (23.5–81.1)	0.156
Urinary Sodium, mEq/L, median (IQR)	60.1 (37.2–89)	72.3 (52.7–86.8)	45.3 (31.1–71.3)	0.091
Urinary Chloride, mEq/L, median (IQR)	52.7 (23.1–94.5)	62.1 (49.1–85.7)	40.8 (22.9–63.1)	0.157
Hemoglobin, g/dL, median (IQR)	13.2 (11.9–14.4)	13.2 (12.3–14.2)	13.4 (11.7–14.6)	0.949
Echocardiographic parameters				
LVEF, %, median (IQR)	39 (31–53.5)	48 (35–64)	39.5 (30.8–51.3)	0.278
LVEDD, mm, median (IQR)	55 (51–61)	49 (42–58)	57 (49.5–62)	0.053
TAPSE, mm, median (IQR)	17.9 (14.3–20)	17 (14–18)	16.3 (14–19)	0.779
PSAP, mmHg, median (IQR)	30 (25–40)	33.5 (29–38.5)	48.5 (37–58.8)	0.003
TAPSE/PAPs ratio, median (IQR)	0.48 (0.37–0.79)	0.54 (0.37–0.61)	0.36 (0.25- 0.49)	0.013
MR grade III-IV, *n* (%)	12 (31.6)	1 (7.7)	15 (41.7)	0.080
TR grade III-IV, *n* (%)	6 (15.8)	2 (15.4)	15 (42.9)	0.020

Continuous variables are expressed as mean ± SD or median (IQR) as appropriate. Abbreviations: BMI, body mass index; COPD, chronic obstructive pulmonary disease; CKD, chronic kidney disease; UACR, urine albumin-to-creatinine ratio; HF, heart failure; ACEI, angiotensin-converting enzyme inhibitor; ARB, angiotensin receptor blocker; ARNI, angiotensin receptor–neprilysin inhibitor; MRA, mineralocorticoid receptor antagonist; SGLT2i, sodium–glucose cotransporter 2 inhibitor; GLP-1, glucagon-like peptide-1 receptor agonist; GDMT, guideline directed medical therapy, eGFR, estimated glomerular filtration rate; AST, aspartate aminotransferase; ALT, alanine aminotransferase; LDH, lactate dehydrogenase; ALP, alkaline phosphatase; GGT, gamma-glutamyl transferase; NT-proBNP, N-terminal pro–B-type natriuretic peptide; CA125, cancer antigen 125; LVEF, left ventricular ejection fraction; LVEDD, left ventricular end-diastolic diameter; TAPSE, tricuspid annular plane systolic excursion; PSAP, pulmonary systolic arterial pressure; MR, mitral regurgitation; TR, tricuspid regurgitation; IQR, interquartile range; SD, standard deviation.

**Table 2:** Clinical outcomes according to groups.

	Group 1 *N* = 39 (44.3%)	Group 2 *N* = 13 (14.8)	Group 3 *N* = 36 (40.9)	*p* Value
HF readmission < 30 days, *n* (%)	3 (7.7)	1 (7.7)	5 (13.9)	0.641
HF readmission/visit emergency department due to HF, *n* (%)	7 (17.9)	2 (15.4)	18 (50)	0.005
Non cardiovascular hospitalization, *n* (%)	19 (48.7)	8 (61.5)	26 (72.2)	0.115
All-cause death, *n* (%)	1 (2.6)	2 (15.4)	6 (16.7)	0.106
HF death, *n* (%)	0	0	6 (16.7)	0.010
Composite endpoint HF readmission or all-cause mortality, *n* (%)	7 (17.9)	4 (30.8)	18 (50)	0.013

## Data Availability

All data generated or analyzed during this study are included in this article and its Supplementary Material files. Further enquiries can be directed to the corresponding authors.

## Supplementary Materials

The following supporting information can be downloaded at: https://www.mdpi.com/article/10.3390/jcm15155825/s1, Table S1: Clinical characteristics according to composite endpoint (HF readmission or all-cause mortality) during follow-up; Table S2: Multivariable Cox regression analysis for the prediction of HF hospitalizations during follow-up according to UACR trajectory groups; Table S3: Multivariable Cox regression analysis for the prediction of the composite endpoint during follow-up according to UACR trajectory groups; Table S4. Bootstrap internal validation of the multivariable Cox models (1000 resamples); Table S5: Sensitivity analysis excluding patients with baseline macroalbuminuria (>300 mg/g at hospital discharge); Table S6: Sensitivity analysis evaluating the association between alternative UACR reduction thresholds and clinical outcomes.
