# Supplementary material for: Early Post-Discharge Albuminuria Trajectory and Clinical Outcomes After Acute Heart Failure
Source: J Clin Med. 2026 Jul 25;15(15):5825. doi: 10.3390/jcm15155825 (PMC13466637; doi:10.3390/jcm15155825)
Supplement: Supplementary file 1 [file jcm-15-05825-s001.zip › jcm-4427799-supplementary.pdf]

Supplementary Table S1: Clinical characteristics according to composite endpoint (HF readmission or all-cause mortality) during follow-up.

	No composite endpoint N = 59 (67)	Composite endpoint N = 29 (33)	P value
Women, n (%)	25 (42.4)	13 (44.8)	0.827
Age, mean ± sd	68.1 ± 13.1	74.5 ± 10.1	0.041
BMI, mean ± sd	27.1 ± 5.5	26.4 ± 6.2	0.570
**Comorbidities**			
Hypertension, n (%)	40 (67.8)	24 (82.8)	0.139
DM, n (%)	23 (39)	18 (62.1)	0.041
Dyslipidaemia, n (%)	30 (50.8)	23 (79.3)	0.010
COPD, n (%)	1 (1.7)	5 (17.2)	0.007
Atrial fibrillation, n (%)	23 (39)	17 (58.6)	0.082
CKD, n (%)	18 (30.5)	15 (51.7)	0.053
**Clinical characteristics**			
Ischemic etiology, n (%)	11 (18.6)	10 (34.5)	0.101
Previous HF admission, n (%)	13 (22)	17 (58.6)	< 0.001
Right or mixed congestion phenotype, n (%)	21 (35.6)	16 (55.2)	0.080
Thiazides / acetazolamide / hypertonic saline use during admission, n (%)	7 (11.9)	9 (31)	0.028
**Treatment at discharge**			
Beta-blockers, n (%)	55 (93.2)	23 (79.3)	0.053
IECA / ARA II, n (%)	15 (25.4)	4 (13.8)	0.213
ARNI, n (%)	47 (79.4)	11 (37.9)	< 0.001
MRA, n (%)	44 (74.6)	20 (69)	0.579
SGLT2i, n (%)	56 (94.9)	24 (82.8)	0.062
GLP1 receptor agonists, n (%)	3 (5.1)	2 (6.9)	0.730
Loop diuretics, n (%)	47 (79.7)	29 (100)	0.009
Dose Loop diuretics, mean ± sd	30.9 ± 23.6	51.7 ± 33.2	0.005
Thiazide use, n (%)	3 (5.1)	3 (10.3)	0.357
GDMT + LVEF < 50%, n (%)	30 (68.2)	8 (50)	0.196
**Laboratory parameters at discharge**			
Urea, mg/dl, median (IQR)	54 (42 - 77)	65 (45 - 96)	0.042
Creatinine, mg/dl, median (IQR)	1.14 (0.91 - 1.37)	1.23 (0.92 - 1.91)	0.124
eGFR, ml/min, median (IQR)	62 (47 - 79)	45 (29 - 71)	0.064
Sodium, mEq/L, median (IQR)	137 (136 - 139)	138 (136 - 140)	0.927
Potassium mEq/L, median (IQR)	4.56 (4.17 - 4.89)	4.45 (4.20 - 4.95)	0.945
Chloride mEq/L, median (IQR)	100 (97 - 102)	98 (96 - 101)	0.170
AST, U/L, median (IQR)	23.5 (17.3 - 33)	20.5 (15.8 - 25.3)	0.077
ALT, U/L, median (IQR)	20 (14 - 29.8)	17.5 (12.8 - 26.3)	0.290
LDH, U/L, median (IQR)	229 (199 - 276)	236 (203 - 272)	0.975
Bilirubin, mg/dl, median (IQR)	0.54 (0.38 - 0.69)	0.43 (0.35 - 0.69)	0.631
ALP, U/L, median (IQR)	83 (63 - 94)	87 (66 - 128)	0.436
GGT, U/L, median (IQR)	31 (19 - 51.5)	26 (18.5 - 64)	0.978
Troponin T, ng/ml, median (IQR)	54.5 (22.3 - 85)	57.5 (30 - 224)	0.322
NT-proBNP, pg/ml, median (IQR)	1725 (757 - 4368)	2928 (1395 - 10959)	0.011
CA 125, U/ml, median (IQR)	44.3 (28.4 - 138)	72.3 (30.5 - 149)	0.559
Urinary Sodium, median(IQR)	54.3 (29.9 - 69.6)	34.6 (24 - 60.2)	0.213
Urinary Chloride, median (IQR)	40.7 (23.2 - 57.4)	28.7 (16.3 - 44)	0.093
UACR, mg/g, median (IQR)	14.8 (8.7 - 43)	64 (11 - 101)	0.050
Hemoglobin, g/dl, median (IQR)	12.8 (11 - 14.5)	11.3 (9.8 - 13.6)	0.057
**Laboratory parameters two weeks post-discharge**			
Urea, mg/dl, median (IQR)	48 (38.5 - 63)	73 (40 - 98)	0.028
Creatinine, mg/dl, median (IQR)	1.12 (0.96 - 1.27)	1.34 (1.03 - 2.04)	0.039
eGFR, ml/min, median (IQR)	61 (47.5 - 76.5)	47 (28 - 66)	0.007
Sodium, mEq/L, median (IQR)	140 (138 - 142)	139 (136 - 141)	0.142
Potassium, mEq/L, median (IQR)	4.67 (4.25 - 4.94)	4.46 (4.19 - 5.08)	0.845
Chloride, mEq/L, median (IQR)	102 (98 - 103)	101 (96 - 103)	0.317

AST, U/L, median (IQR)	22 (18.3 - 27)	22 (15 - 29)	0.543
ALT, U/L, median (IQR)	18 (14 - 26.5)	16 (9 - 20)	0.045
LDH, U/L, median (IQR)	208 (179 - 252)	213 (196 - 257)	0.280
Bilirubin mg/dl, median (IQR)	0.47 (0.34 - 0.70)	0.61 (0.42 - 0.81)	0.180
ALP, U/L, median (IQR)	92 (74 - 108)	92 (68.8 - 138)	0.469
GGT, U/L, median (IQR)	27 (18 - 64)	28 (19.5 - 54.3)	0.778
NT-proBNP, pg/ml, median (IQR)	1415 (750 - 3192)	3375 (1640 - 7745)	< 0.001
CA 125 U/ml, median (IQR)	31.7 (17.3 - 74.1)	38.2 (21.9 - 72.8)	0.417
Sodium Urinary, mEq/L, median (IQR)	66.9 (44.7 - 93)	39.9 (28.8 - 61.4)	0.001
Chloride Urinary, mEq/L, median (IQR)	61.8 (35.8 - 94)	32 (20.3 - 57.8)	0.005
UACR mg/g, median (IQR)	14.4 (7.4 - 33.8)	50 (23.9 - 189)	< 0.001
Hemoglobin g/dl, median (IQR)	13.5 (12 - 14.8)	12.4 (11.2 - 14)	0.033
**Echocardiographic parameters**			
LVEF, %, median (IQR)	38 (30 - 49)	48 (35 - 59)	0.083
< 40% LVEF, n (%)	35 (59.3)	11 (37.9)	0.138
41-50% LVEF, n (%)	9 (15.3)	5 (17.2)	0.811
> 50% LVEF, n (%)	15 (25.4)	13 (44.8)	0.066
LVEDD, mm, median (IQR)	58 (49 - 61)	52.5 (48 - 59.5)	0.252
TAPSE mm, median (IQR)	16.8 (13.7 - 19)	18 (15 - 20)	0.068
PASP, mmHg, median (IQR)	31 (25 - 50)	44 (37 - 58)	0.002
TAPSE / PASP ratio, median (IQR)	0.47 (0.35 - 0.71)	0,40 (0.26 - 0.50)	0.072
Mitral regurgitation grade III-IV, n (%)	17 (28.8)	11 (39.3)	0.329
Tricuspid regurgitation grade III-IV, n (%)	12 (20.3)	11 (40.7)	0.047

Continuous variables are expressed as mean ± SD or median (IQR) as appropriate. Abbreviations: BMI, body mass index; SD, standard deviation; DM, diabetes mellitus; COPD, chronic obstructive pulmonary disease; CKD, chronic kidney disease; HF, heart failure; ACEI, angiotensin-converting enzyme inhibitors; ARB, angiotensin receptor blockers; ARNI, angiotensin receptor–neprilysin inhibitor; MRA, mineralocorticoid receptor antagonists; SGLT2i, sodium–glucose cotransporter 2 inhibitors; GLP-1, glucagon-like peptide-1; GDMT, guideline-directed medical therapy; eGFR, estimated glomerular filtration rate; AST, aspartate aminotransferase; ALT, alanine aminotransferase; LDH, lactate dehydrogenase; ALP, alkaline phosphatase; GGT, gamma-glutamyl transferase; NT-proBNP, N-terminal pro-B-type natriuretic peptide; CA125, cancer antigen 125; UACR, urine albumin-to-creatinine ratio; LVEF, left ventricular ejection fraction; LVEDD, left ventricular end-diastolic diameter; TAPSE, tricuspid annular plane systolic excursion; PASP, pulmonary artery systolic pressure; MR, mitral regurgitation; TR, tricuspid regurgitation.

Supplementary Table S2: Multivariable Cox regression analysis for the prediction of HF hospitalizations during follow-up according to UACR trajectory groups

Variable	Multivariable analysis (Full model)						Multivariable analysis (Optimal model)				
	**n**	**b**	**EE**	**HR**	**CI(95%)**	**p-value**	**b**	**EE**	**HR**	**CI(95%)**	**p-value**
Groups: Group 1	86			1 (ref)					1 (ref)		
Groups: Group 2	86	-0.098	0.873	0.907	0.16 - 5.02	0.911	0.09	0.82	1.094	0.22 - 5.46	0.913
Groups: Group 3	86	0.863	0.532	2.37	0.84 - 6.73	0.105	1.128	0.488	3.089	1.19 - 8.04	0.021
HBP: Yes	86	-0.506	0.641	0.603	0.17 - 2.12	0.43	-	-	-	-	-
DM: Yes	86	0.634	0.464	1.885	0.76 - 4.68	0.172	-	-	-	-	-
Dyslipidaemia: Yes	86	0.321	0.579	1.378	0.44 - 4.28	0.58	-	-	-	-	-
CKD: Yes	86	0.431	0.524	1.430	0.51 – 4.03	0.496					
Previous HF admission: Yes	86	1.358	0.466	3.889	1.56 - 9.7	0.004	1.41	0.428	4.096	1.77 - 9.48	<0.001
LVEF.admission	86	0.026	0.014	1.026	1 - 1.06	0.068	0.026	0.012	1.026	1 - 1.05	0.034
MR_grade.III.IV: Yes	86	0.198	0.497	1.22	0.46 - 3.23	0.69	-	-	-	-	-
TR_grade.III.IV: Yes	86	0.198	0.464	1.219	0.49 - 3.03	0.67	-	-	-	-	-
Mix / Right congestion phenotype: Yes	86	-0.002	0.435	0.998	0.43 - 2.34	0.997	-	-	-	-	-
Concordance (C-index)	0.8145						0.794				

Supplementary Table S3: Multivariable Cox regression analysis for the prediction of the composite endpoint during follow-up according to UACR trajectory groups.

Variable		Multivariable analysis (Full model)					Multivariable analysis (Optimal model)				
	**n**	**b**	**EE**	**HR**	**CI (95%)**	**p-value**	**b**	**EE**	**HR**	**CI (95%)**	**p-value**
Groups: Group 1	86			1 (ref)					1 (ref)		
Groups: Group 2	86	0.517	0.799	1.677	0.35 - 8.02	0.518	0.489	0.745	1.63	0.38 - 7.03	0.512
Groups: Group 3	86	1.037	0.571	2.82	0.92 - 8.63	0.069	1.198	0.529	3.314	1.18 - 9.35	0.024
HBP: Yes	86	-0.239	0.701	0.787	0.2 - 3.11	0.733	-	-	-	-	-
DM: Yes	86	0.716	0.468	2.046	0.82 - 5.12	0.126	0.681	0.442	1.976	0.83 - 4.7	0.124
Dyslipidaemia: Yes	86	0.312	0.609	1.366	0.41 - 4.5	0.609	-	-	-	-	-
CKD: Yes	86	0.413	0.598	1.160	0.45 – 2.99	0.759					
Previous HF admission: Yes	86	1.289	0.457	3.628	1.48 - 8.89	0.005	1.33	0.441	3.781	1.59 - 8.97	0.003
LVEF at admission	86	0.032	0.014	1.032	1 - 1.06	0.028	0.03	0.012	1.031	1.01 - 1.06	0.014
MR.grade.III.IV: Yes	86	0.362	0.515	1.437	0.52 - 3.94	0.481	-	-	-	-	-
TR.grade.III.IV: Yes	86	0.215	0.466	1.239	0.5 - 3.09	0.645	-	-	-	-	-
Mix / Right congestion phenotype: Yes	86	0.14	0.436	1.151	0.49 - 2.71	0.748	-	-	-	-	-
Concordance (C-index)	0.828						0.824				

Supplementary Table S4. Bootstrap internal validation of the multivariable Cox models (1000 resamples).

	**Composite endpoint**	**HF hospitalization**
Events, n	29	27
Parameters, k	5	4
Events per variable (EPV)	5.8	6.8
Apparent C-index	0.778	0.762
Optimism-corrected C-index	0.746	0.729
Optimism	0.032	0.033
Shrinkage factor	0.821	0.783

Composite endpoint model included trajectory group, age, previous HF hospitalization, and LVEF. HF hospitalization model included trajectory group, previous HF hospitalization, and LVEF. Optimism-corrected C-index and shrinkage factor were derived using Harrell's bootstrap validation procedure.

Supplementary Table S5: Sensitivity analysis excluding patients with baseline macroalbuminuria (>300 mg/g at hospital discharge).

Outcome	Primary analysis HR (95% CI); *p*	Sensitivity analysis HR (95% CI); *p*
HF hospitalization	3.28 (1.28–8.45); p = 0.014	2.92 (1.10–7.74); p = 0.032
Composite endpoint (HF hospitalization or all-cause mortality)	3.00 (1.18–7.64); p = 0.021	2.61 (1.00–6.83); p = 0.050

Abbreviations: HR = Hazard ratio ; CI = confidence Interval.

Supplementary Table S6: Sensitivity analysis evaluating the association between alternative UACR reduction thresholds and clinical outcomes.

UACR reduction threshold	Group 2	Group 3	Composite endpoint HR (95% CI)	p	HF hospitalization HR (95% CI)	p
30%	17	32	2.80 (1.08–7.26)	0.034	3.05 (1.16–7.99)	0.023
40%	15	34	2.94 (1.14–7.54)	0.025	3.20 (1.23–8.32)	0.017
50% (primary analysis)	13	36	3.00 (1.18–7.64)	0.021	3.28 (1.28–8.45)	0.014
60%	13	36	3.00 (1.18–7.64)	0.021	3.28 (1.28–8.45)	0.014
70%	12	37	3.13 (1.23–7.93)	0.016	3.46 (1.35–8.85)	0.010

*Hazard ratios correspond to Group 3 versus Group 1 (reference category). Group 2 was not significantly associated with either endpoint across any threshold.

Abbreviations: HR = Hazard ratio ; CI = confidence Interval.
